# Genome-Wide Identification of the Expansin Gene Family and Its Potential Association with Drought Stress in Moso Bamboo

**DOI:** 10.3390/ijms21249491

**Published:** 2020-12-14

**Authors:** Kang-Ming Jin, Ren-Ying Zhuo, Dong Xu, Yu-Jun Wang, Hui-Jin Fan, Bi-Yun Huang, Gui-Rong Qiao

**Affiliations:** 1 State Key Laboratory of Tree Genetics and Breeding, Chinese Academy of Forestry, Beijing 100091, China; 17858287379@163.com (K.-M.J.); zhuory@gmail.com (R.-Y.Z.); xudongzhuanyong@163.com (D.X.); yujunwang0618@foxmail.com (Y.-J.W.); fhj1201@163.com (H.-J.F.); hby948750582@163.com (B.-Y.H.); 2 Key Laboratory of Tree Breeding of Zhejiang Province, The Research Institute of Subtropical of Forestry, Chinese Academy of Forestry, Hangzhou 311400, China

**Keywords:** expansins, evolutionary pattern, abiotic stress, plant cell wall, drought

## Abstract

Expansins, a group of cell wall-loosening proteins, are involved in cell-wall loosening and cell enlargement in a pH-dependent manner. According to previous study, they were involved in plant growth and abiotic stress responses. However, information on the biological function of the expansin gene in moso bamboo is still limited. In this study, we identified a total of 82 expansin genes in moso bamboo, clustered into four subfamilies (α-expansin (EXPA), β-expansin (EXPB), expansin-like A (EXLA) and expansin-like B (EXPB)). Subsequently, the molecular structure, chromosomal location and phylogenetic relationship of the expansin genes of *Phyllostachys edulis* (*PeEXs)* were further characterized. A total of 14 pairs of tandem duplication genes and 31 pairs of segmented duplication genes were also identified, which may promote the expansion of the expansin gene family. Promoter analysis found many cis-acting elements related to growth and development and stress response, especially abscisic acid response element (ABRE). Expression pattern revealed that most *PeEXs* have tissue expression specificity. Meanwhile, the expression of some selected *PeEXs* was significantly upregulated mostly under abscisic acid (ABA) and polyethylene glycol (PEG) treatment, which implied that these genes actively respond to expression under abiotic stress. This study provided new insights into the structure, evolution and function prediction of the expansin gene family in moso bamboo.

## 1. Introduction

Bamboo is one of the most important non-timber forestry products which is characterized by fast growth with a long vegetative period and high yield, and its forest production has been estimated at about USD 2.5 billion per year on average [1]. Because of its high degree of lignification, tenacity and evergreen bamboo leaves, bamboo has become a kind of special forest resources [2].

Moso bamboo (*Phyllostachys edulis*), a typical non-timber forest member, is a bamboo species with long cultivation and the widest area and the most important economic value in China. Additionally, the culm of moso bamboo grows fast and has a large biomass. In particular, it takes only 24 h to grow up more than 1 m in the period of the fastest growth [3]. Therefore, moso bamboo plays important roles in economy, ecology, culture, aesthetics and technology. Moreover, the root of moso bamboo is a fibrous root system, which enters the soil shallowly. Once extreme dry weather occurs, it will cause the death of bamboo forests. Drought also reduces the decomposition process, leading to the accumulation of organic matter on the forest floor, thereby increasing the frequency or intensity of fires or reducing nutrient cycling [4]. This will cause great harm to yield and ecological environment.

Expansins are cell wall-related proteins that are involved in cell wall loosening and cell enlargement in a pH-dependent manner [5] and are involved in plant growth and abiotic stress responses according to relevant studies [6,7,8,9,10,11,12,13,14,15,16,17,18,19,20,21]. The first expansins were found in an experiment of acid-induced extension in cucumber hypocotyl [22]. In the plant, expansins were involved in the relaxation of the cell wall, which release the mechanical tension of the cell wall polymer by breaking the non-covalent bond that binds the microfilament and cause some polysaccharides to slide under turgor pressure, thus causing the cell wall to loosen and promote cell extension [23]. Subsequently, expansins were widely found in growing plant tissues and mature fruits [24,25]. Related proteins are also found in non-plant organisms such as bacteria, fungi, molluscs and nematodes that may help digest plant biomass [26,27].

A typical expansin consists of 250–275 amino acids, which consists of two domains and a signal peptide with a length of 20–30 amino acid residues at the N-terminal [28]. Domain 1 is a double-psi beta-barrel (DPBB), which consists of 120–135 amino acid residues. Its core sequence has high homology with the catalytic domain of glycoside hydrolase family 45 proteins (GH45). This region is cysteine-rich, which may be related to the formation of disulfide bonds. Domain 2 is composed of 90-120 amino acid residues, which has certain homology with group-2 grass pollen allergen protein (G2A) of *Gramineae* [5,28]. This sequence is conserved and is tryptophan-rich, whose microenvironment is extremely sensitive to changes in pH. It has been shown that a reduction in pH can lead to great changes in the molecular conformation of the enzyme [29], which will consequently affect the function of expansins.

According to phylogenetic analysis [30] and standardized nomenclature [31], they can be divided into four subfamilies: α-expansin (EXPA), β-expansin (EXPB), expansin-like A (EXLA) and expansin-like B (EXLB). In terms of function and quantity, the EXPA and EXPB families are the two main subfamilies in plants. Functional studies have shown that expansins are involved in plant growth and development, such as promoting plant tissue and cell growth [6], seed development [7], root hair initiation [8,9,10] and root growth [11,12], leaf and stem development [13,14], pollen tube elongation [15,16], petiole shedding [13], fruit ripening [17] and so on. Expansins also play a vital role in abiotic stress response, such as to drought and heat stress [18,19,20,21]. There is experimental evidence that EXPA and EXPB proteins are required for the cell extension and developmental processes of cell wall modification [30]. In addition, the EXPA family is a highly conserved protein family which is related to the elongation of cell wall and mainly exists in non-gramineous dicotyledonous plants and monocotyledons [32]. The EXPB family mainly exists in *Gramineae* monocotyledons, and its biological effect may be to soften the stigma and accelerate the pollen tube to reach the ovule through the maternal tissue [33], whereas EXLA and EXLB families only have two typical conserved domains, with far less known about their exact function [14]. Notably, *BrEXLB1*, under its specific promoters, has been shown to be involved in the regulation of leaf and plant growth and responds to hormone availability, light quality, dark periods, developmental stages and drought conditions in transgenic *Arabidopsis* [34].

To date, expansins have been identified in the cell walls of nearly 100 species which were widespread in dicotyledons and monocotyledons, such as *Arabidopsis thaliana* and rice [30], maize [35], wheat [36], poplar [37] and so on. To our knowledge, however, very limited information is available regarding the expansin gene family in moso bamboo. Recently, the available moso bamboo genome [3,38] and the bamboo genome database (BambooGDB) [39] have provided us with an opportunity to perform a genome-wide analysis of the expansin gene family in moso bamboo. In this study, we identified 82 expansin genes from the *P. edulis* genome. Moreover, we systematically analyzed detailed information on the exon–intron structure, motif compositions, chromosomal locations and the cis-acting elements in their promoters. In addition, the phylogenetic relationships among the expansin genes in *Arabidopsis thaliana*, *Oryza sativa*, *Populus trichocarpa* and *P. edulis* were also compared. We further gave the evolutionary patterns and expression analysis of the expansin genes. This study provided valuable insights into the functional characterization of the expansin gene family members in moso bamboo.

## 2. Results

### 2.1. Identification of the Expansin Genes in Moso Bamboo

In this study, 82 expansin genes with two conserved domains (DPBB_1 and Pollen_allerg_1 domains) were identified in the *P. edulis* genome by HMMsearch. According to the previous standardized nomenclature, all expansin genes were divided into four subfamilies and named sequentially on the basis of their chromosomal position. Similar to *Arabidopsis thaliana*, rice, poplar and other plants, the EXPA subfamily composed the largest clade with 45 members, followed by 29 EXLB, 7 EXPB and 1 EXLA member(s) (Table 1). The identified full-length expansin genes of *Phyllostachys edulis (PeEXs)* encoded proteins with relative molecular weights (MWs) from 22,440.46 (*PeEXPB8*) to 53,579.25 (*PeEXLA1*) Da and length ranges from 170 (*PeEXPA37*) to 506 (*PeEXLA1*) amino acids (aa) and contained secretory signal peptides. In addition, it was predicted that the isoelectric point (pI) of expansins ranged from 4.82 (*PeEXPB8*) to 9.73 (*PeEXPA27*). The results of subcellular localization suggested that all *PeEXs* were just located on the cell wall, except for *PeEXLA1* and *PeEXPA27*, using the online software Plant-mPLoc [40]. Table S1 shows the details of the identified expansins.

### 2.2. Phylogenetic Analysis of Expansins in the Four Different Plant Species, Distribution of Expansin Genes on Scaffolds

**Figure 1:**
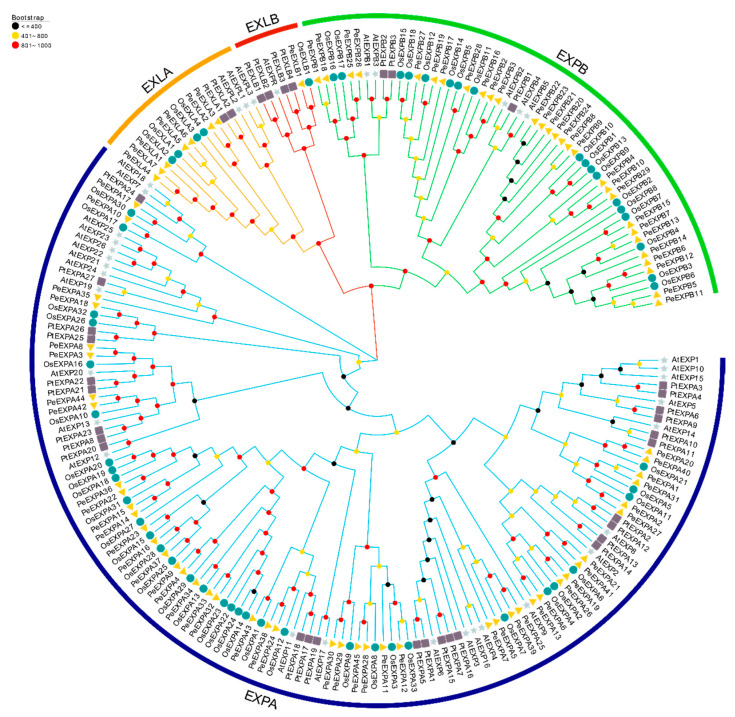
Phylogenetic tree of expansins from moso bamboo, rice, *Arabidopsis* and poplar. MEGA7.0 was used to construct a neighbor-joining (NJ) phylogenetic tree with 1000 bootstrap replications. Triangles, circles, stars and squares represent the expansins of moso bamboo, rice, *Arabidopsis* and poplar, respectively. Different-colored circles on the branches represent bootstrap values.

**Figure 2:**
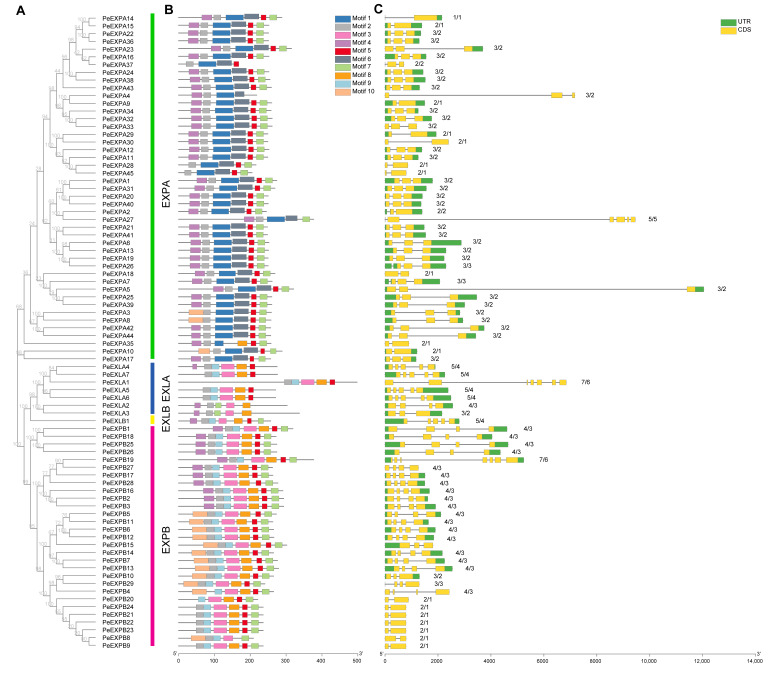
Phylogenetic relationships, motif compositions and gene structure of expansin genes in moso bamboo. (**A**) Phylogenetic relationships of 82 expansins in moso bamboo. They were classified into four groups: α-expansin (EXPA), β-expansin (EXPB), expansin-like A (EXLA) and expansin-like B (EXPB); (**B**) different motif compositions of expansins in moso bamboo were detected using MEME. The conserved motifs are represented by boxes with different colors and their information is listed in Table 2; (**C**) gene structure organization of expansin genes of *Phyllostachys edulis* (*PeEXs*). The number of introns and exons is also marked in the figure.

In order to further understand the phylogenetic relationship among the expansin gene family members in moso bamboo, rice, poplar and *Arabidopsis thaliana*, MEGA7.0 was used to construct a neighbor-joining (NJ) phylogenetic tree with 1000 bootstrap replications. As shown in the phylogenetic tree (Figure 1), the expansin genes of different species were apparently divided into four subfamilies: EXPA, EXPB, EXLA and EXLB. Members of the four subfamilies gathered separately. Through the phylogenetic trees of moso bamboo (Figure 2), 29 homologous gene pairs were found, among which 25 homologous gene pairs had high bootstrap values (>90%). In addition, the genetic relationship of the same subfamily genes among different species can be approximately estimated on the phylogenetic tree, and the evolution process of expansin genes in various plants can be further analyzed. Numerous members from different plant species, such as *OsEXLB1* and *PeEXLB1*, *PeEXPA34* and *OsEXPA13*, were clustered in the same small subfamily, which indicated that the expansin genes have a close evolutionary relationship between moso bamboo and rice. This result also supported the argument that expansin genes evolved from a common ancestor.

**Figure 3:**
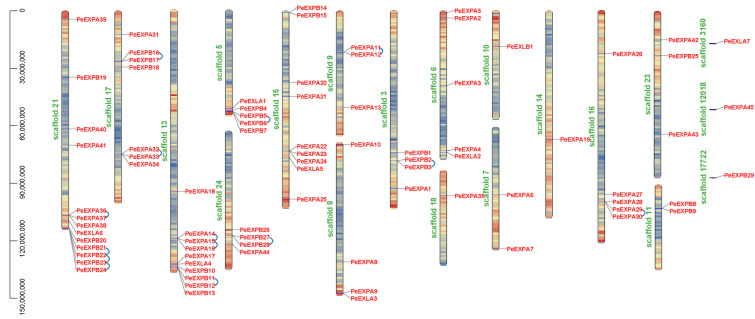
Distribution of expansin genes on scaffolds in moso bamboo. The gene density is displayed in the form of heat maps on the scaffolds; high and low gene density are represented by red and blue, respectively, and 14 tandem duplication pairs are represented by the blue lines.

The results of chromosomal location showed that *PeEXs* were distributed unevenly on the scaffolds of moso bamboo, and the number of expansin genes on every putative scaffold was quite different due to the incomplete genome and low genetic variation abundance of moso bamboo. According to the chromosome mapping (Figure 3), scaffold 21 contains the largest number (13) of expansin genes, followed by scaffold 13 and scaffold 15, with 10 and 9 expansin genes, respectively. However, only one expansin gene was found on scaffold 10, scaffold 14, scaffold 18, scaffold 3160, scaffold 12,018 and scaffold 17,722. The number of expansin genes on the rest of scaffolds varied from 2 to 7. Interestingly, *PeEXLB1*, which was the only member of the EXLA subfamily, was located on scaffold 10. No other expansin genes can be found in this scaffold, which was unique in this result. In addition, we found the largest number of genes on scaffold 10, including 6 EXPA, 6 EXPB and 1 EXLA subfamily member(s). At the same time, tandem duplication gene pairs, which are considered to be an important driving force for the occurrence of new biological functions, existed on some scaffolds, such as *PeEXPA36* and *PeEXPA37*, *PeEXPA32* and *PeEXPA33*, etc. Notably, there were four pairs of tandem duplication gene pairs on scaffold 21 (Figure 3).

### 2.3. Gene Structure, Conserved Protein Motifs of PeEXs

In the gene family, the importance of the distribution pattern of the number of introns and exons is obvious. It is regarded as the skeleton and evolutionary fingerprint of the gene, which can be used to understand the structural evolution of the family. We provide the gene structure organization in Figure 2; most members of the same subfamily exhibited similarities in structure, showing consistency. For instance, most members (68.9%) of the EXPA subfamily contained three exons, while other members usually had two or five exons. However, the gene structure of the EXLB subfamily exhibited 5 exons and 4 introns, similar to the EXLA subfamily. The EXPB subfamily can be divided into two groups according to the number of exons: one had two exons and the other had four exons. The reason for these differences may be the loss or acquisition of exons induced by long-term evolution. In addition, there are multiple introns in the EXLA subfamily; in particular, *PeEXLA1* presented the largest number of exons (six). The other genes had no significant difference, showing consistency, and each subfamily contained more than one intron. This suggested that there is a tight evolutionary relationship between members in the same subfamily.

The MEME Web server was used to study the motif diversity in the translation of our putative *PeEXs*. The obtained MEME motif information is shown in Table 2, which is named from motif 1 to motif 10 in turn. The results (Figure 2) showed that the type, number and order of motifs in the same subfamily are almost alike; on the contrary, there were some divergences among different subfamilies, but there was also a certain contact. Noticeably, the EXPA subfamily was the largest branch which had definitely stable motif compositions. Except for *PeEXPA3*, *PeEXPA8*, *PeEXPA10*, *PeEXPA28*, *PeEXPA37* and *PeEXPA45*, the motifs of the remaining members were highly conservative, indicating that they may perform similar functions in moso bamboo. However, the EXPB subfamily presented two typical motif structures—one containing motif 4 and the other containing motif 10, both of which contained motifs 2, 3, 5, 7, 8 and 9, while the motif structure of the former member was entirely similar to the EXLB subfamily. It indicated that the EXLB subfamily evolved from a branch of the EXPB subfamily, and there was a certain functional relationship. In addition, the three subfamilies, EXPB, EXLA and EXLB, all had motifs 2, 3, 5, 8 and 9. This implied that these subfamilies had a close evolutionary and phylogenetic relationship. Generally speaking, motifs 2, 5, and 7 were relatively conservative in the expansins of moso bamboo, and almost all the proteins encoded by *PeEXs* had these three kinds of motifs.

### 2.4. Detection of Cis-Regulatory Elements in the Promoter Regions of PeEXs

**Figure 4:**
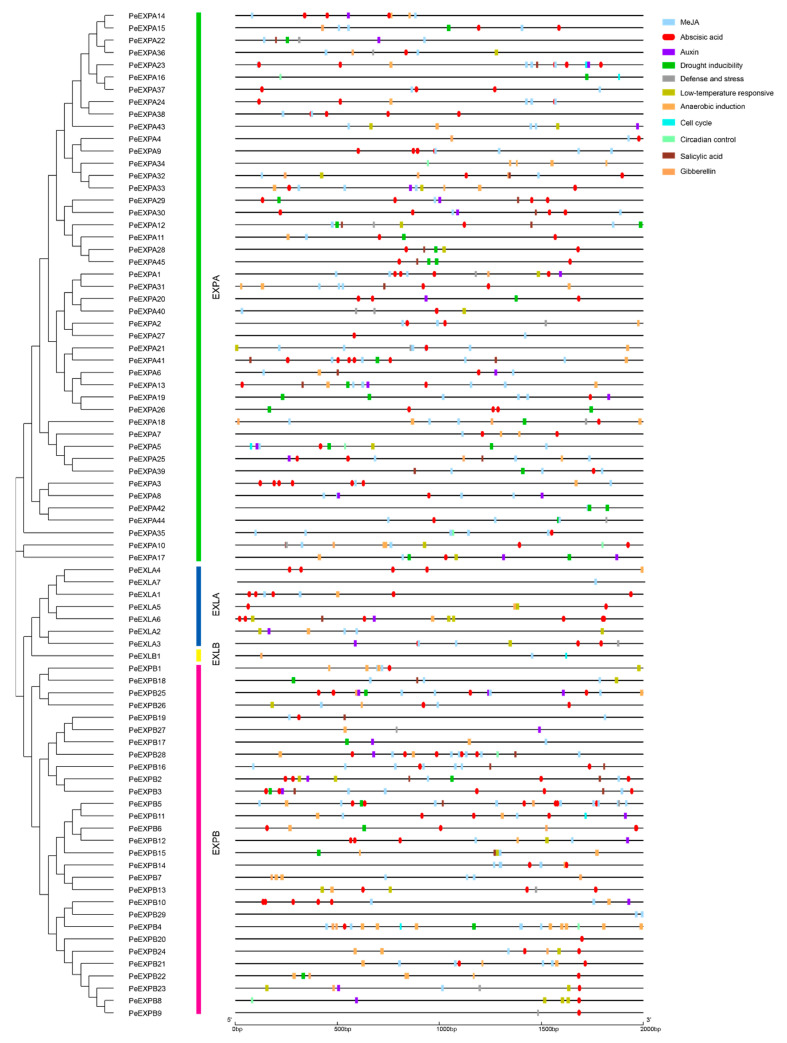
Predicted cis-elements of expansin gene promoters in moso bamboo. Promoter regions 2000 bp upstream of 82 *PeEXs* analyzed by PlantCARE. Different-colored rectangles represented different cis-elements, and ABA response elements are highlighted in red ellipses.

**Figure 5:**
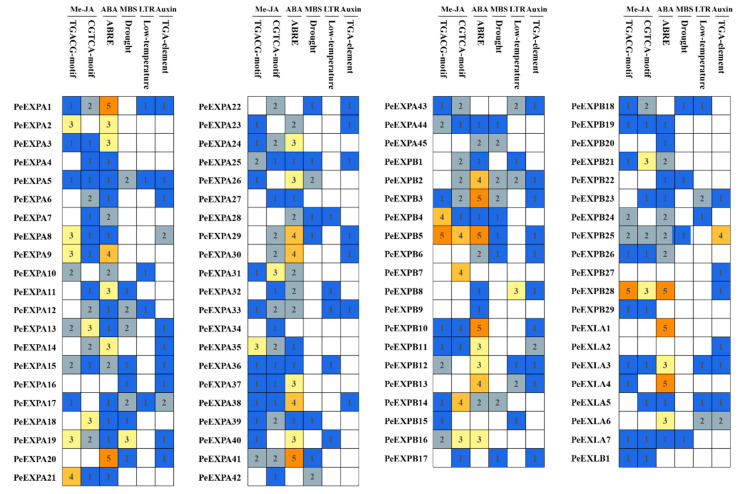
The numbers of promoter elements in the *PeEXs’* promoter regions. Five major cis-elements of *PeEXs* are indicated by different-colored boxes with numbers.

To further understand the potential function and regulatory mechanism of *PeEXs*, we analyzed the promoter region of the upstream 2000 bp of 82 putative *PeEXs*, which plays an important role in gene function and regulation. A total of 13 cis-regulatory elements, such as light response elements, abscisic acid (ABA) response elements, MeJA stress response elements, auxin response elements and other abiotic stress response elements, were detected in this study. The results are shown in Figure 4. Interestingly, in the promoter region of 82 putative *PeEXs*, the majority of them had ABA response elements. Additionally, ABREs and MeJA stress response elements were found in the promoter regions of 69 and 68 *PeEXs*, respectively. With regard to abiotic stress response elements, we found an MYB binding site involved in drought inducibility (MBS) and low-temperature response elements (LTR) in the promoter regions of 31 and 23 *PeEXs*, respectively. Among them, *PeEXPA5*, *PeEXPA17*, *PeEXPA18*, *PeEXPB2* and *PeEXPB18* had both MBS elements and LTR elements in the promoter region, which indicated that these genes play an important role in the abiotic stress response of moso bamboo. We counted the number of major cis-elements in the promoter region for further functional analysis (Figure 5). Numerous expansins had more than three ABREs in the promoter region, such as *PeEXPA1*, *PeEXPA3*, *PeEXPA9*, *PeEXPA11*, *PeEXPA29* and so on. It was worth noting that a total of 2 MBS elements and 5 ABREs were found in the promoter region of *PeEXPB3*, and 1 MBS element, 4 ABREs and some other abiotic stress response elements were found in the promoter region of *PeEXPA29*. As these genes had a high number of cis-regulatory elements related to ABA and drought stress responses, they were selected for further analysis to obtain their detailed functions and regulatory mechanisms.

### 2.5. Synteny Analysis

**Figure 6:**
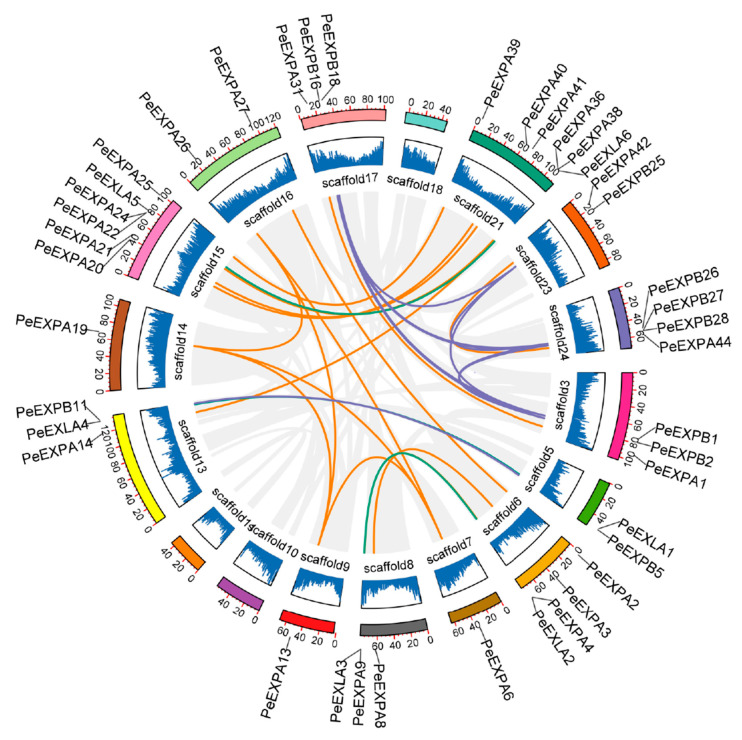
Syntenic analysis of expansin genes in moso bamboo. Different scaffolds are shown in different colors. The gene density is displayed in the form of histogram. The inner gray lines represent all the collinearity relationships in moso bamboo, and the inner orange, purple and green lines represent the collinearity relationships of EXPA, EXPB and EXLA subfamilies, respectively. A total of 31 pairs of segmented duplication genes were detected in the *P. edulis* genomes.

We used MCScanX to detect the synteny regions in the *P. edulis* genomes to further investigate the evolutionary relationship of the expansin gene family. A total of 31 collinear gene pairs were obtained in moso bamboo. The lines with different colors represent the collinear gene pairs of different subfamilies, the gray lines represent the whole collinear gene pairs presenting in the *P. edulis* genome and the histogram shows the gene density on scaffolds (Figure 6). Obviously, except for scaffold 10, scaffold 11 and scaffold 18, each scaffold had collinear gene pairs. It can be seen that the majority of collinear relationships occur on scaffolds with a higher gene density. In addition, the number of collinear gene pairs between scaffold 15 and scaffold 21 was the largest, and most of them belonged to the EXPA subfamily. It indicated that the expansion of the expansin gene family may occur through large-scale chromosome segmental duplication in moso bamboo.

### 2.6. Evolutionary and Divergence Patterns of the Expansin Genes

In the process of gene evolution, the frequent occurrence of gene duplication events will lead to the emergence of orthologous and paralogous pairs. Through phylogenetic tree analysis, 12 putative orthologous gene pairs (*Pe–Os*) and 19 putative paralogous gene pairs (*Pe–Pe*) were identified with high bootstrap values. Table 3 lists all orthologous and paralogous pairs. In order to analyze the evolution and divergence pattern of expansins, the Ks values and Ka/Ks ratios of all putative orthologous and paralogous pairs were calculated. According to previous studies, the Ks value can be used as a substitute of time to roughly estimate large-scale duplication events through the formula T = Ks/2λ.

**Figure 7:**
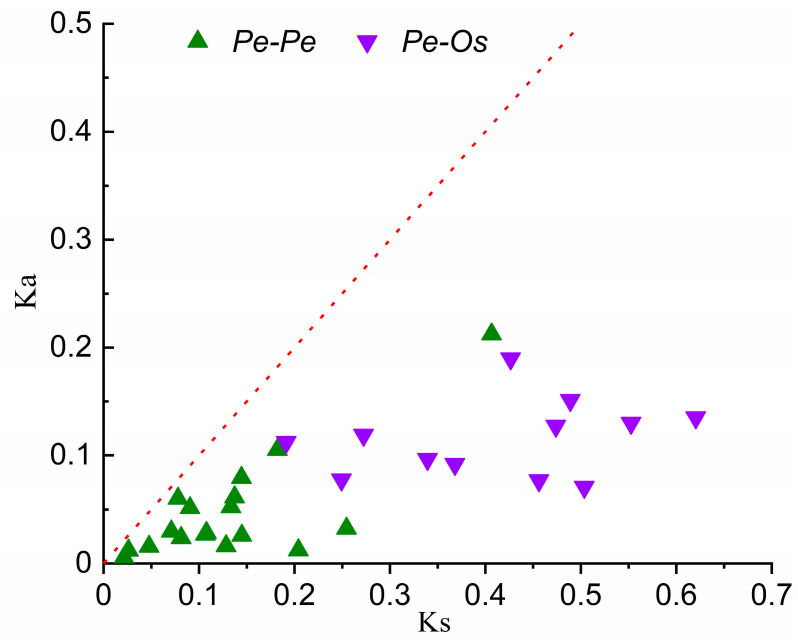
Distribution of Ka and Ks from paralogous (*Pe–Pe*) and orthologous (*Pe–Os*) gene pairs. Twelve putative orthologous gene pairs (*Pe–Os*) and nineteen putative paralogous gene pairs (*Pe–Pe*) are displayed in different colors and shapes, and the red dashed line indicates the slope of Ka/Ks = 1.

As shown in Figure 7, the Ks value of the paralogous pairs (*Pe–Pe*) reached a peak at 0.10–0.15, which indicates that the *PeEXs* underwent a large-scale duplication event about 7–12 million years ago (Mya), forming the paralogous pairs of moso bamboo. Interestingly, Peng et al. (2013) speculated that whole-genome duplication occurred about 7–12 Mya, based on the analysis of the whole-genome duplication event of moso bamboo [3]. Our results are similar to this report and support that *PeEXs* were expanding as the whole genome duplicated. The Ks value of orthologous pairs (*Pe–Os*) reached a peak at about 0.40–0.50, indicating that the divergence time of *PeEXs* and expansin genes of *Oryza sativa* (*OsEXs)* was about 31–38 Mya. According to the results of a previous study, the divergence time of moso bamboo and rice was approximately 7–15 Mya, which indicated that the expansin gene underwent genetic evolution before the separation of the two ancestral species. Generally speaking, a Ka/Ks ratio greater than 1 indicates that a gene has experienced positive selection, which will be beneficial to adaptive genetic variation; a Ka/Ks ratio equal to 1 indicates neutral selection, while a Ka/Ks ratio less than 1 indicates negative selection or purifying selection, which reduces the change rate of amino acids. The Ka/Ks ratios of divergent genes were all less than 1, which implied that the expansin gene between moso bamboo and rice genomes and the paralogous genes in moso bamboo genome had undergone strong purifying selection.

### 2.7. qRT-PCR Expression Analysis of the Expansin Genes in Moso Bamboo

**Figure 8:**
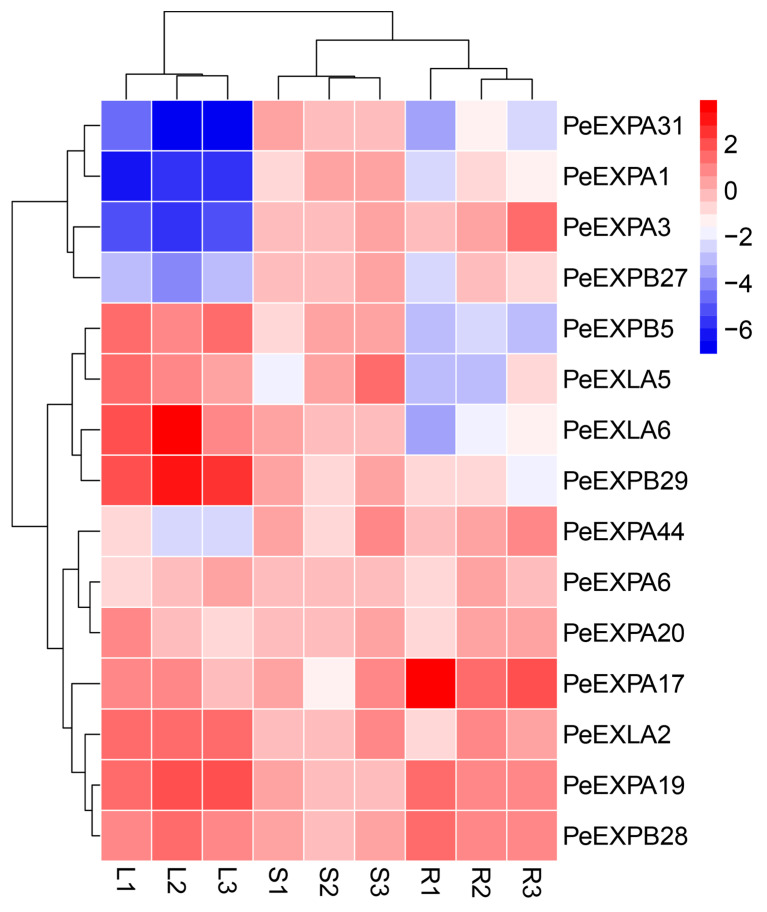
Expression pattern of 15 selected *PeEXs* in different tissues. The heat map was drawn using log2 logarithmic transformed expression values. Red and blue represent high and low expression levels, respectively. Based on the expression, the expansin genes were hierarchically clustered and divided into various gene clusters in the figure. L, leaves; S, stems; R, roots; 1, 2 and 3 represent the three biological replicates. Three repetitions were used to reduce technical errors when performing qRT-PCR.

To obtain the expression pattern of the expansin gene family in different tissues, we selected 15 expansin genes based on promoter analysis. The qRT-PCR analysis showed that *PeEXs* have tissue expression specificity, except *PeEXPA6* and *PeEXPA20* (Figure 8 8 and Figure S2). *PeEXPA1*, *PeEXPA3*, *PeEXPA31*, and *PeEXPB27*, under normal conditions, were all low expressed in leaves. *PeEXLA5*, *PeEXLA6*, *PeEXPB5* and *PeEXPB29* were highly expressed in leaves, but low expressed in roots. *PeEXPA19* and *PeEXPB28* were evidently highly expressed in roots and leaves. However, *PeEXLA2* and *PeEXPA17* were preferentially expressed in leaves and roots, respectively. In general, there were significant differences in the expression level of the most of expansin genes in leaves, shoots and roots.

**Figure 9:**
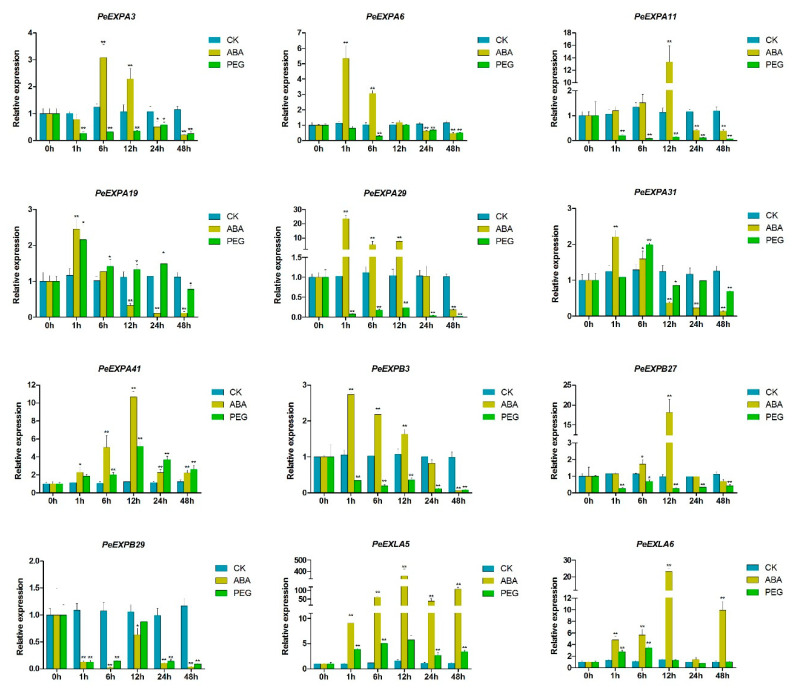
qRT-PCR expression analysis of 12 selected *PeEXs* under abscisic acid (ABA) and polyethylene glycol (PEG) treatments. The expression levels of untreated (0 h) group were normalized to 1 as a control. CK, untreated samples. Error bars were obtained from three biological replicates. Statistically significant differences between the expression level of control group and treatment group were analyzed by Duncan’s test, which are represented by asterisks. * *p* < 0.05; ** *p* < 0.01.

Promoter analysis showed that most *PeEXs* possess ABA response elements and the MYB binding site involved in drought inducibility. Therefore, the 12 selected expansin genes were tested by qRT-PCR to verify whether their expression levels would be induced under ABA treatment and drought conditions simulated by PEG treatment (Figure 9).

Under ABA treatment, except for *PeEXPB29*, the remaining 11 selected expansin genes were prominently upregulated and were then downregulated. Particularly, *PeEXPA29*, *PeEXPB27*, *PeEXLA5* and *PeEXLA6* showed an intense response to ABA at 12 h after treatment compared with at 0 h. In addition, the expression levels of *PeEXPA6*, *PeEXPA19*, *PeEXPA29*, *PeEXPA31* and *PeEXPB3*, respectively possessing 1, 1, 4, 2 and 5 ABA response elements, peaked at 1 h after treatment, which indicated that they rapidly respond to the ABA signal.

PEG treatment was used to simulate drought stress conditions to stimulate the expression of plant defense genes. Under the treatment, *PeEXPA19*, *PeEXPA31*, *PeEXPA41*, *PeEXLA5* and *PeEXLA6* were significant upregulated and subsequently downregulated, whereas the remaining expansin genes exhibited downregulation at all time points compared with at 0 h. Furthermore, the peak expression of *PeEXPA31*, *PeEXLA5* and *PeEXLA6* was discovered at 6 h. Interestingly, *PeEXPA19*, *PeEXPA31*, *PeEXPA41*, *PeEXPB29*, *PeEXLA5* and *PeEXLA6* showed semblable expression trends in different treatments. Collinear genes *PeEXLA5* and *PeEXLA6* showed highly similar expression patterns under the two treatments, indicating that their biological functions also have a certain similarity. These results were generally consistent with the aforementioned differential distribution of cis-elements in the promoter region of *PeEXs*.

### 2.8. Co-Expression Analysis of the Expansin Genes in Moso Bamboo

**Figure 10:**
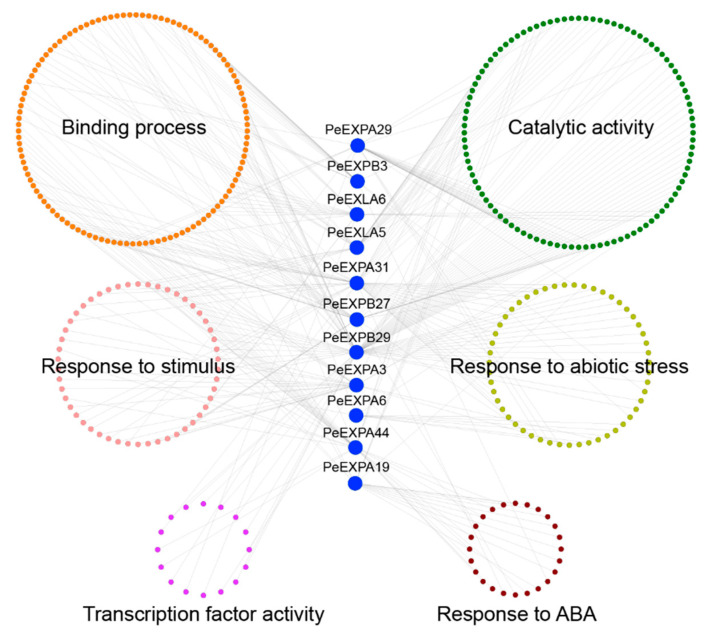
Co-expression regulatory network of expansin genes in moso bamboo. These genes are divided into the following six kinds by GO function annotation in different colors: binding process, catalytic activity, response to stimulus, response to abiotic stress, transcription factor activity and response to ABA.

In this study, we reconstructed the co-expression regulatory network of 11 selected genes using the BambooNET online database (Figure 10). There were 366 nodes and 448 connections in the co-expression network. The GO annotation classification results showed that the majority of the co-expressed genes were enriched in biological pathways, such as binding process (116 edges), catalytic activity (103 edges), biological regulation (16 edges), stimulus response (98 edges), abiotic stress response (89 edges) and ABA response (26 edges), which implied an important function of expansins in response to stimuli and abiotic stresses in plants. Among them, *PeEXPB29* has 79 nodes, which is the largest module in the network, including 16 nodes involving binding processes, 28 nodes involving catalytic activity, 10 nodes involving stimulus response, 16 nodes involving abiotic stress response and 9 nodes involving transcription factor activity. *PeEXPA31*, *PeEXPB29* and *PeEXPA6* were distinctly closely connected with genes related to abiotic stress response. *PeEXPA19* had the most nodes related to ABA response, which also echoed the above results.

## 3. Discussion

The plant cell wall is a unique cell structure of plant cells. To a large extent, it determines the shape of plant cells, controls the direction and rate of cell growth and affects the differentiation and functional realization of all kinds of cells [42]. The formation of cell wall includes the relaxation and reconstruction of cell wall and the synthesis, assembly and deposition of cell wall components. Among them, the relaxation of cell wall has a profound effect on plant development, which has received great attention in recent years [5,43]. Drought stress will affect plant turgor pressure and extensibility of cell wall, which are critical elements in regulating plant cell growth and driving cell wall expansion. Eventually, a series of changes in plant morphology will occur because of cell wall modification [44,45]. Playing a central role in regulating extensibility was a group of cell wall-related proteins which adjust the loosening and expansion of cells [46]. Hence, analyzing the biological functions and molecular mechanisms of these proteins will facilitate to obtain a deeper understanding of the pathways adapting to environmental pressures during plant growth.

Expansins, commonly found in plants, are cell wall related-proteins that are involved in cell wall loosening. In this study, 82 expansin genes with two conserved domains were identified in the *P. edulis* genome through a genome-wide analysis. Similar to other plants, all putative *PeEXs* were divided into four subfamilies: EXPA, EXPB, EXLA and EXLB (Table 1). Each subfamily has 45, 29, 7 and 1 members, respectively. Obviously, the expansin gene family has undergone a large expansion in moso bamboo (82), and its numbers of members were far more than in *Arabidopsis thaliana* (36), rice (56) and poplar (36), which was mainly reflected in the EXPB subfamily. In addition, we mapped putative *PeEXs* to the corresponding scaffold based on the location information of the *P. edulis* genome (Figure 3). The results showed that *PeEXs* were distributed unevenly on the scaffolds of moso bamboo. Further analysis showed that the expansins exhibit certain subfamily characteristics in intron/exon patterns, motif structures and phylogenetic relationships (Figure 2). This high evolutionary conservation can be used as an important basis for subfamily classification.

Generally, tandem duplication, large-scale chromosome segmental duplication and transposition were identified as the main evolutionary mechanisms that cause the expansion of gene family [47]. A total of 14 pairs of tandem duplication genes and 31 pairs of segmented duplication genes were identified exactly, which may promote the expansion of the expansin gene family (Figure 3 and Figure 6). Moreover, evolutionary and divergence analysis showed that the expansin gene family formed paralogous pairs (*Pe–Pe*) through a large-scale genome-wide duplication event around 7–12 Mya [3], which greatly expanded the gene family in moso bamboo. Compared with *Arabidopsis* and rice, the expansin gene family has been greatly expanded in moso bamboo, which may indicate that the participation of *PeEXs* in some specific biological processes is crucial. The special effects of these genes under growth and development and abiotic stress may explain the high biomass, rapid growth and other unique traits of moso bamboo, although there may be functional redundancy of some genes. Moreover, the divergence between *PeEXs* and *OsEXs* occurred in an earlier period, about 31–38 Mya, when the ancestral species of bamboo and rice had not yet separated. The Ka/Ks results also manifested that 12 orthologous gene pairs and 19 paralogous gene pairs have undergone strong purifying selection during evolution (Figure 7).

The results of the qRT-PCR analysis showed that most *PeEXs* had tissue expression specificity (Figure 8). For instance, *PeEXLA5*, *PeEXLA6*, *PeEXPB5*, *PeEXPA19* and *PeEXPB29* were highly expressed in leaves, while *PeEXPA3*, *PeEXPA17*, *PeEXPA44* and *PeEXPB28* had root expression preference. In general, continuous root growth is essential to accommodate to various resistances in in the plant, and expansins happen to play an active participant in this process. Expansin genes were highly expressed at all stages of wheat root development, especially in seedling roots [36]. In *Arabidopsis thaliana*, the expression of *AtEXP7* and *AtEXP18* can regulate the initiation of root hairs, and RNA interference with the expression of *AtEXP7* will shorten root hairs oppositely [48]. *PeEXPA17* was closely related to *AtEXP7* and *AtEXP18* in the phylogenetic tree, suggesting that *PeEXPA17* may play a positive role in root growth. In addition, *OsEXPB2* also was a root-predominant gene which played a crucial role in root hair formation [49]. Furthermore, expansins also play an indispensable role in plant organogenesis and other growth processes. *OsEXPA8* can promote suspension cell division and growth and increased the cell number and size in rice [50]. *AtEXPA1*, an *Arabidopsis* guard cell-expressed expansin, was deemed to regulate stomatal opening by altering the structure of the guard cell wall [51]. Fruit ripening and organ shedding also correlated with expansins. Overexpression of tomato *LeEXP1* gene softens the fruit at the time of green fruit, but if its expression reduces, the fruit is still hard after ripening [52]. In many other plants, softening of fruit ripening has also been identified to be related to specifically expressed expansins, such as strawberry [53], cherry [54] and peach [55]. In particular, a series of key candidate genes related to the rapid growth of bamboo were given in the transcriptome analysis of the slow-growing mutants of moso bamboo. A total of 36 expansion protein genes were found to be differentially expressed, of which 10 were upregulated relative to WT plants and 26 showed downregulation of expression [56]. In other words, the lack of the participation of expansins will greatly affect the growth and development of plants.

ABA, as a stress signal, is essential during plant growth and development. It integrates various stress signals and controls downstream stress responses to make plants adapt to various stress environments through uninterrupted adjustments [57]. Promoter analysis showed that almost all *PeEXs* have ABA response elements, especially *PeEXPA1*, *PeEXPA20*, *PeEXPB5* and *PeEXLA4*, which all have five ABRE promoter elements (Figure 5). The 11 selected *PeEXs* were regulated by the ABA signaling pathway and their expression levels were significantly upregulated in this study, and the expression levels of five selected *PeEXs* were significantly upregulated under PEG stress conditions, which indicated that these genes may actively participate in related signal pathways under abiotic stress (Figure 9). It is worth noting that some *PeEXs* showed similar expression trends under the two treatments, indicating that their response to drought stress may depend on the ABA signaling pathway. Numerous studies also support the role of expansins in plant stress resistance. TaMPS, the MYB transcription factor of wheat root, will directly bind with the promoter of *TaEXPA2* to regulate its expression, thus making a positive contribution to drought tolerance [19]. Equally, *PeEXPA19*, the homologous gene of *TaEXPA2*, was also significantly upregulated under drought stress, and it possessed 3 MBS elements. Previous reports indicated that *OsEXPA10* may be involved in a variety of signal transduction pathways that mediate biological defenses and play a balanced role between growth and development and biotic resistance in rice [58]. *PeEXPA44*, the homologous gene of *OsEXPA10*, exhibited downregulated expression in ABA and PEG treatment (Figure S1). Whether *PeEXPA44* has a balance mechanism between growth and abiotic stress still needs further evidence in moso bamboo. Under drought stress, the transcriptional activity of expansin can be increased in the apical 5 mm of maize primary roots, which can enhance the extensibility of the cell wall and help root cells to maintain elongation while reducing turgor pressure [33]. Overexpression of the *PttEXPA8* gene can improve plant stress resistance to high temperature, drought, cold and salt stress to varying degrees in transgenic tobacco plants [59]. Meanwhile, co-expression analysis results demonstrated that the role of expansin genes was irreplaceable in stimulus response and abiotic stress response (Figure 10). Especially, *PeEXPA19* appeared to be closely related to the ABA response, which was consistent with promoter and expression analysis. In other words, the above results uniformly proved that *PeEXs* plays a vital role in plant abiotic stress.

## 4. Conclusions

Overall, this study gave the first genome-wide identification and analysis to obtain the molecular characterization, evolutionary pattern and biological function of the 82 expansin genes in the *P. edulis* genome. More importantly, these results showed that expansin genes play a significant role in abiotic stress and plant growth. In this paper, the comprehensive study of expansin genes is conducive to the selection of suitable candidate genes, and further analysis of the mechanism of stress resistance in moso bamboo is required.

## 5. Materials and Methods

### 5.1. Genome-Wide Identification of Expansin Genes

To identify the expansin genes, the genome and protein sequences of moso bamboo were downloaded from the BambooGDB database (http://www.bamboogdb.org/). We used two different approaches to identify the members of the expansin gene family in moso bamboo [41,60]. First, 35 expansin sequences from *Arabidopsis thaliana* were obtained from EXPANSIN CENTRAL (http://www.personal.psu.edu/fsl/ExpCentral/index.htm), which were used as query sequences to perform blast searches against the genome and protein sequences of moso bamboo with a 1 × 10^−5^ cut-off E value. Then, all protein sequences of putative expansin genes were examined by SMART (http://smart.embl-heidelberg.de/) and PFAM (http://pfam.xfam.org/) to confirm the existence of the conserved domain, and all of the expansin genes without DPBB_1 domain and Pollen_allerg_1 domain were discarded. Second, we used the Hidden Markov Model (HMM) with PFAM numbers PF03330 and PF01357 to search putative expansin genes in our protein dataset using HMMSEARCH with a threshold of e-values < 10^−5^ [61]. Finally, we manually checked these proteins to ensure that all had expansin protein domains. The molecular weight (MW) and isoelectric point (pI) were acquired from the ExPaSy (https://web.expasy.org/compute_pi/) for each protein sequence, and the online software Plant-mPLoc (http://www.csbio.sjtu.edu.cn/bioinf/plant-multi/#) was used to predict their subcellular localization.

### 5.2. Exon–Intron Structure, Conserved Motif, Genic Physical Location on Scaffolds and Cis-Regulatory Elements Analysis

The exon–intron structures were mapped using TBtools [62] according to the obtained coding sequence (CDS) and genomic sequences of moso bamboo. Conserved motifs present in the expansins were identified with the online MEME tool (http://meme-suite.org/tools/meme) using the default parameter settings: maximum number of motifs = 10, whose results were shown by TBtools. In addition, each expansin gene was mapped to the scaffolds according to its coordinates on the genome using TBtools. Through the Quick MCScanX Wrapper program of TBtools, we obtained the tandem duplication gene pairs of the expansin gene family in moso bamboo. Meanwhile, we examined the 2000-bp upstream sequences of the expansin genes to identify the cis-elements in the putative promoter regions by SPDE [63].

Plant CARE (http://bioinformatics.psb.ugent.be/webtools/plantcare/html/) was used to identify the predicted cis-regulatory elements present in the gene promoters related to stress responses and hormone effects, and these results were visualized by TBtools.

### 5.3. Phylogenetic Tree Construction

ClustalW was used for multiple sequence alignment to explore the evolutionary relationships of expansins between moso bamboo, rice, poplar and *Arabidopsis* with default parameters [64], and MEGA 7.0 was used to construct a neighbor-joining (NJ) phylogenetic tree in the four different plant species with the following parameters: NJ tree method, complete deletion and bootstrap analysis with 1000 replicates [65].

### 5.4. Synteny and Gene Duplication Analyzes of the Expansin Genes in Moso Bamboo

We analyzed the duplication events of the expansin genes by MCScanX [66] with the default parameters. Circos was used to create collinear analysis diagrams based on information about collinear pairs and genetic location [67]. Calculating the non-synonymous (Ka) replacement rate and synonymous (Ks) rate between collinear pairs can be used to estimate the selective pressure on the dataset and the occurrence of duplication events. Then, a Ka/Ks calculator was used to analyze gene duplication events to calculate non-synonymous substitution rate and synonymous replacement rate [68]. Using the Ks value as a rough estimate of the time of large-scale duplication events [69], Ks is transformed into a time framework using the common formula T = Ks/2λ × 10^−6^ Mya [70]. According to the previous estimate of the synonymous replacement rate, the clock style variable (λ) was designated as 6.5 × 10^−9^ years for both moso bamboo and rice [3]. The above operations were done using TBtools.

### 5.5. Co-Expression Network Construction

The co-expression relationships of 11 selected genes were obtained through the BambooNET database (http://bioinformatics.cau.edu.cn/bamboo/) [71], and then, the co-expressed genes were classified according to the GO annotation information. Finally, the co-expression regulatory network was performed using Cytoscape software.

### 5.6. Plant Materials, Growth Conditions and Stresses Treatment

Moso bamboo seeds were germinated on filter paper at a certain humidity and then grown in half-strength Hoagland–Arnon solution at 25 °C (16 h light, 8 h dark) in an artificial climate incubator. Four-week-old seedlings were treated with 10 µM abscisic acid (ABA) and 10% PEG8000 at similar growth statuses [72]. Approximately 2-cm long roots were collected from the seedlings after treatment for 0, 1, 6, 12, 24 and 48 h. In order to analyze the tissue-specific expression patterns of expansins, the leaves, roots and stems of 6-week-old seedlings were also collected at similar growth status. Additionally, we used the untreated group at the same time point as a control to exclude the effect of time. Three biological and three technical replicates were performed. All samples were frozen in liquid nitrogen immediately and then stored at −80 °C for RNA isolation.

### 5.7. RNA Isolation and Quantitative RT-PCR

Total RNA was extracted from above-mentioned samples using the Tiangen RNAprep plant kit (Tiangen). We used RNase-free DNase1 to remove genomic DNA contamination before dissolving RNA (Tiangen). Additional PCR reactions and agarose gel electrophoresis were used to recheck the purity of RNA. Then, we used the Takara PrimeScript First-Strand cDNA Synthesis kit (Takara) to synthesize first-strand cDNA. All first-strand cDNA samples were diluted 5 times and stored at −20 °C for real-time quantitative PCR (qRT-PCR) experiments. All specific quantitative primers were designed by Primer Premier 5.0 and are shown in Table S2. Real-time quantitative reverse transcription PCR was performed using the TB Green™ Premix Ex Taq™ (Tli RNaseH Plus) kit (Takara) with the QuantStudio™ 7 Flex Real-Time PCR instrument (Applied Biosystems). A 20-µL reaction system was used, and each reaction contained 0.4 µL gene-specific primers, 0.4 µL ROX Reference Dye, 2 µL cDNA sample, 6.8 µL ddH_2_O and 10 µL TB Green Premix Ex Taq reagent. The relative expression level was evaluated based on the 2^−ΔΔCT^ method, and tonoplast intrinsic protein 41 (*TIP41*) gene was used as the reference gene [73].

## Figures and Tables

**Table 1:** Number of expansin genes in five plant species.

Species	EXPA	EXPB	EXLA	EXLB	Total	References
*Arabidopsis*	26	6	3	1	36	[30]
Rice	33	18	4	1	56	[30]
Poplar	27	3	2	4	36	[37]
Apple	34	1	2	4	41	[41]
Moso bamboo	45	29	7	1	82	

**Table 2:** Detailed information of motifs.

Motif	Length	Protein Sequence	PFAM Domain
1	50	VTVTATNFCPPNYALPSDBGGWCNPPRQHFDMAZPAFEKIAIYRAGIVPV	DPBB_1
2	21	ALSTALFNDGAGCGACYZIRC	
3	37	HFDLSGTAFGAMAKPGKADQLRHAGIIDIQFRRVPCK	
4	29	HATFYGGSDASGTMGGACGYGNLYSQGYG	
5	15	WTPMSRNWGANWQSB	
6	41	RRVPCVRKGGIRFTINGHSYFELVLVTNVAGAGDVASVSVK	Pollen_allerg_1
7	21	FRVTSSDGRTLVANBVIPAGW	
8	29	FHVEKGSNPNYLAVLVEYEDGDGDIVAVD	Pollen_allerg_1
9	21	TKPEACSGEPVTVVITDMNYE	
10	41	GKWLAAKATWYGAPTGAGPDDNGGACGFKBVNQPPFSSMTS	

**Table 3:** Orthologous (*Pe–Os*) and paralogous (*Pe–Pe*) expansin gene pairs.

*Pe–Os*	*Pe–Pe*
*PeEXPB15/OsEXPB7*	*PeEXPA20/PeEXPA40*
*PeEXPB28/OsEXPB5*	*PeEXPA2/PeEXPA27*
*PeEXPB19/OsEXPB12*	*PeEXPA21/PeEXPA41*
*PeEXLB1/OsEXLB1*	*PeEXPA19/PeEXPA26*
*PeEXPA17/OsEXPA30*	*PeEXPA25/PeEXPA39*
*PeEXPA10/OsEXPA17*	*PeEXPA28/PeEXPA45*
*PeEXPA18/OsEXPA32*	*PeEXPA29/PeEXPA30*
*PeEXPA23/OsEXPA15*	*PeEXPA24/PeEXPA38*
*PeEXPA16/OsEXPA28*	*PeEXPA32/PeEXPA33*
*PeEXPA37/OsEXPA25*	*PeEXPA4/PeEXPA9*
*PeEXPA34/OsEXPA13*	*PeEXPA14/PeEXPA15*
*PeEXPA43/OsEXPA1*	*PeEXPA22/PeEXPA36*
	*PeEXPA42/PeEXPA44*
	*PeEXPA3/PeEXPA8*
	*PeEXLA2/PeEXLA3*
	*PeEXPB25/PeEXPB26*
	*PeEXPB2/PeEXPB3*
	*PeEXPB10/PeEXPB29*
	*PeEXPB6/PeEXPB12*

## Supplementary Materials

The following are available online at https://www.mdpi.com/1422-0067/21/24/9491/s1.

## Abbreviations

PeEXs	Expansin genes of Phyllostachys edulis
OsEXs	Expansin genes of *Oryza sativa*
Mya	Million years ago
ABRE	Abscisic acid response element
G2A	Group-II pollen allergen protein
NJ	Neighbor-joining
PEG	Polyethylene glycol
MeJA	Methyl jasmonate
MYB	Myeloblastosis
Ka	Nonsynonymous substitution rates
Ks	Synonymous substitution rates
GO	Gene ontology
WT	Wild type
TaMPS	Homolog to OsMPS and named according to OsMPS
SMART	Simple modular architecture research tool
PFAM	Database of protein families
MEME	Multiple em for motif elicitation
ROX	ROX reference dye
qRT-PCR	Quantitative real-time polymerase chain reaction
