# Supplementary material for: Genome-Wide Identification of the Expansin Gene Family and Its Potential Association with Drought Stress in Moso Bamboo
Source: Int J Mol Sci. 2020 Dec 14;21(24):9491. doi: 10.3390/ijms21249491 (PMC7764852; doi:10.3390/ijms21249491)
Supplement: Supplementary file 1 [file ijms-21-09491-s001.pdf]

Supplementary Materials:

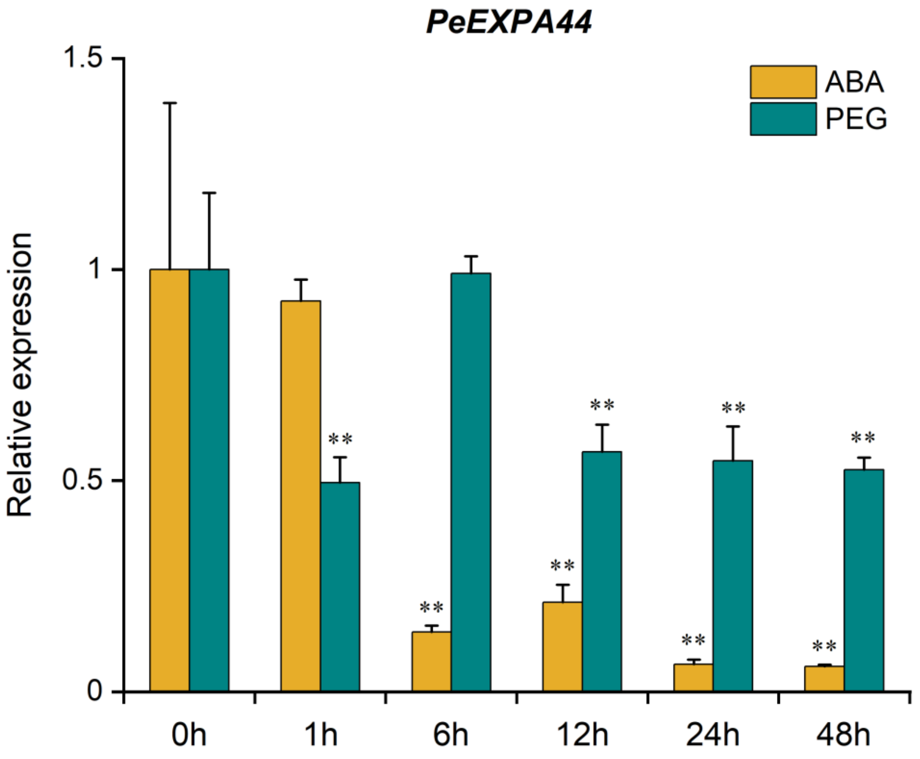

Figure S1 qRT-PCR expression analysis of *PeEXPA44* under ABA and PEG treatments. The expression levels of untreated (0 h) group were normalized to 1 as control. Error bars were obtained from three measurements. Statistically significant differences between the expression level of control group and treatment group were represented by asterisks. * $p < 0.05$; ** $p < 0.01$

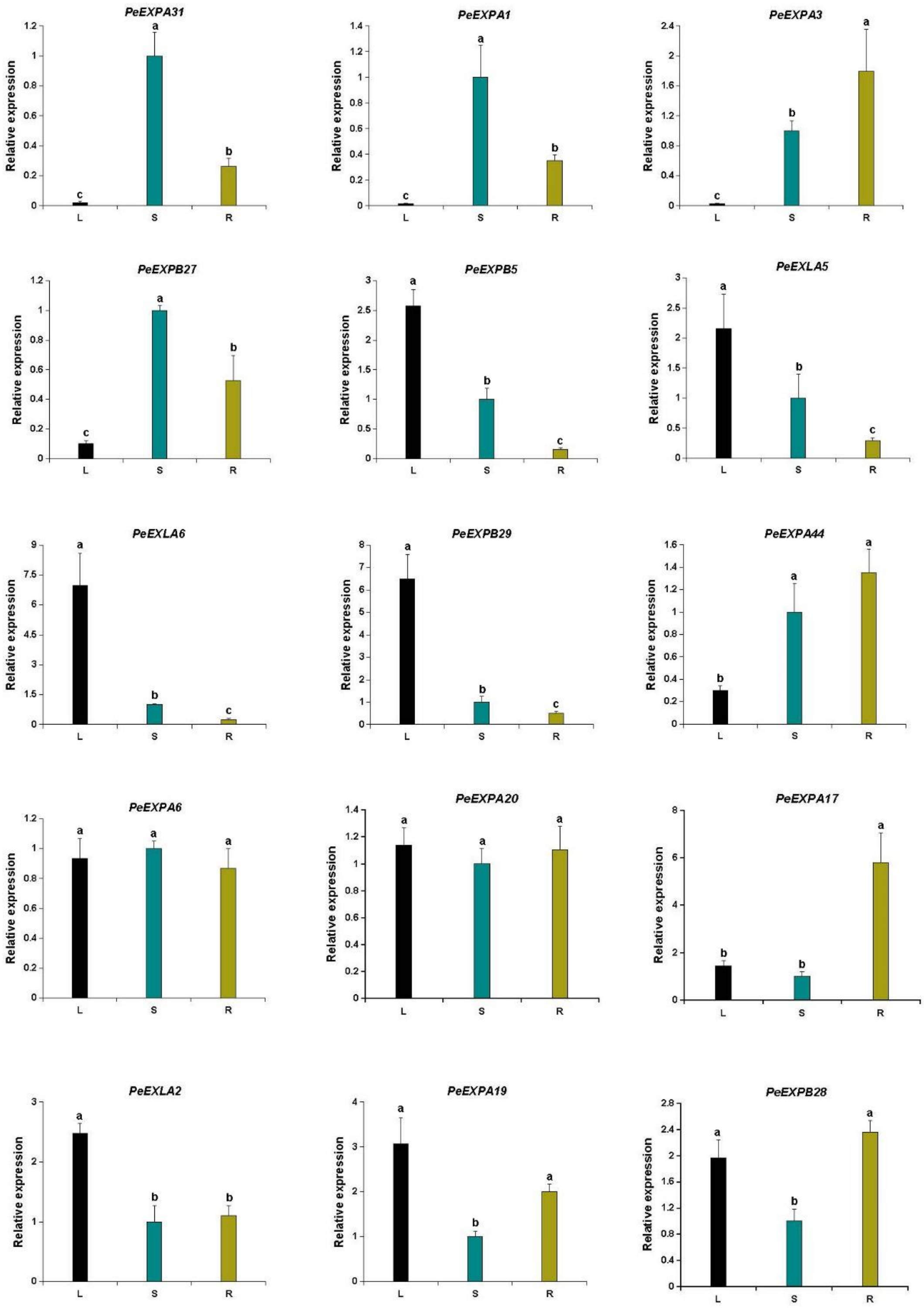

Figure S2. Significant differences in the expression levels of 15 selected PeEXs in different tissues. The significance of differences was analyzed by Duncan's test ($P < 0.05$). Different lowercase letters represent statistically significant differences. Error bars were obtained from three biological replicates. L, leaves; S, stems; R, roots.

Table S1. Details of the identified expansin genes in moso bamboo

Gene Name	Gene ID	Scaffold	start	end	strand	Length (aa)	PI	MW (Da)	Subcellular Location
PeEXPB1	PH02Gene40569.t1	3	74335277	74339262	+	324	9.12	33895.65	Cell wall
PeEXPB2	PH02Gene42691.t1	3	78490857	78492274	+	297	8.70	30644.73	Cell wall
PeEXPB3	PH02Gene45247.t1	3	78537523	78538940	+	297	8.70	30644.73	Cell wall
PeEXPA1	PH02Gene06444.t1	3	92969207	92970218	+	278	9.26	28294.80	Cell wall
PeEXLA1	PH02Gene29510.t1	5	51422163	51429007	+	506	7.12	53579.25	Cell wall. Chloroplast
PeEXPB4	PH02Gene02968.t1	5	52353073	52355499	+	269	9.46	29100.59	Cell wall
PeEXPB5	PH02Gene02937.t2	5	52983207	52985034	-	277	9.10	29823.89	Cell wall
PeEXPB6	PH02Gene02936.t1	5	52999419	53000792	-	269	5.20	28634.17	Cell wall
PeEXPB7	PH02Gene02934.t1	5	53032671	53034490	+	280	8.12	30415.35	Cell wall
PeEXPA2	PH02Gene12214.t2	6	3701059	3701885	+	249	6.02	24982.91	Cell wall
PeEXPA3	PH02Gene46545.t1	6	38936166	38938611	-	263	8.33	28208.14	Cell wall
PeEXPA4	PH02Gene31926.t1	6	73058480	73065647	+	221	8.03	22933.90	Cell wall
PeEXLA2	PH02Gene31603.t1	6	73854562	73856641	+	308	7.93	33129.66	Cell wall
PeEXPA5	PH02Gene47068.t1	6	896945	908588	-	326	9.65	34040.70	Cell wall
PeEXPA6	PH02Gene28974.t1	7	35252681	35254304	-	256	8.07	26590.58	Cell wall
PeEXPA7	PH02Gene23772.t1	7	63371448	63372607	-	265	9.66	27676.46	Cell wall
PeEXPA8	PH02Gene28134.t1	8	62256118	62258592	+	262	7.47	28095.97	Cell wall
PeEXPA9	PH02Gene24828.t1	8	78600808	78601679	+	264	9.28	27952.61	Cell wall
PeEXLA3	PH02Gene29461.t2	8	78769363	78770954	+	342	6.78	37217.27	Cell wall
PeEXPA10	PH02Gene31819.t1	8	987131	988084	-	293	6.16	30742.74	Cell wall
PeEXPA11	PH02Gene18295.t1	9	21565985	21566932	-	253	8.40	26792.07	Cell wall
PeEXPA12	PH02Gene18296.t1	9	21601983	21603082	-	256	8.70	27065.60	Cell wall
PeEXPA13	PH02Gene05526.t1	9	50302323	50303746	-	255	7.55	26517.43	Cell wall
PeEXLB1	PH02Gene13121.t1	10	18732234	18734165	-	261	5.22	27614.94	Cell wall
PeEXPB8	PH02Gene11268.t1	11	12195104	12195898	-	212	4.82	22440.46	Cell wall
PeEXPB9	PH02Gene11271.t1	11	12255991	12256782	-	239	5.64	25697.43	Cell wall
PeEXPA14	PH02Gene14288.t1	13	119228669	119230631	-	292	8.23	31073.25	Cell wall
PeEXPA15	PH02Gene14289.t1	13	119249926	119250818	+	256	6.67	27058.42	Cell wall
PeEXPA16	PH02Gene20872.t1	13	119260590	119261581	-	256	5.54	27291.88	Cell wall
PeEXPA17	PH02Gene22581.t1	13	132305242	132306188	+	261	8.72	27646.48	Cell wall
PeEXLA4	PH02Gene38031.t1	13	132903689	132905428	+	279	9.07	29452.59	Cell wall
PeEXPB10	PH02Gene01623.t1	13	133791899	133792907	-	269	8.97	28855.23	Cell wall
PeEXPB11	PH02Gene01648.t3	13	134369411	134370757	-	267	6.93	28725.48	Cell wall
PeEXPB12	PH02Gene01649.t1	13	134394493	134395901	-	270	5.07	28743.35	Cell wall
PeEXPB13	PH02Gene01650.t1	13	134427766	134429684	+	283	6.38	30624.52	Cell wall
PeEXPA18	PH02Gene36807.t1	13	94700314	94701215	+	273	8.94	28736.69	Cell wall
PeEXPA19	PH02Gene03683.t1	14	67001984	67003518	+	254	6.02	26556.47	Cell wall
PeEXPA20	PH02Gene45046.t1	15	37401407	37402384	-	254	9.50	26538.10	Cell wall
PeEXPA21	PH02Gene46862.t1	15	44591817	44592860	-	252	8.82	26077.39	Cell wall
PeEXPA22	PH02Gene03583.t1	15	73176039	73177057	-	255	6.51	27159.72	Cell wall
PeEXPA23	PH02Gene03585.t1	15	73228018	73231320	-	320	9.27	34235.31	Cell wall
PeEXPA24	PH02Gene03586.t1	15	73255714	73256665	-	256	8.31	26873.12	Cell wall
PeEXLA5	PH02Gene34262.t1	15	76838243	76839604	-	275	8.84	29617.75	Cell wall
PeEXPB14	PH02Gene40297.t1	15	902854	904335	-	269	8.34	28877.80	Cell wall
PeEXPB15	PH02Gene40298.t1	15	911752	913008	+	307	5.16	32261.83	Cell wall

PeEXPA25	PH02Gene42531.t1	15	98226831	98229181	-	264	9.34	27795.50	Cell wall
PeEXPA26	PH02Gene32628.t1	16	22660027	22661286	-	254	5.47	26443.28	Cell wall
PeEXPA27	PH02Gene13181.t1	16	95918380	95927832	-	383	9.73	40209.34	Cell wall. Chloroplast
PeEXPA28	PH02Gene14425.t1	16	99869453	99870316	-	219	9.58	23534.85	Cell wall
PeEXPA29	PH02Gene14424.t1	16	99895265	99896734	-	253	5.55	26308.25	Cell wall
PeEXPA30	PH02Gene50367.t1	16	99927882	99930278	-	253	5.20	26293.23	Cell wall
PeEXPA31	PH02Gene01054.t1	17	12261072	12262082	-	273	9.18	27794.28	Cell wall
PeEXPB16	PH02Gene28532.t1	17	26234003	26235223	-	295	9.03	30540.75	Cell wall
PeEXPB17	PH02Gene28533.t1	17	26300287	26301515	-	267	5.90	27437.88	Cell wall
PeEXPB18	PH02Gene16914.t1	17	29342022	29345539	-	276	8.76	29186.28	Cell wall
PeEXPA32	PH02Gene21084.t1	17	74673666	74674865	-	265	8.99	27993.83	Cell wall
PeEXPA33	PH02Gene38735.t1	17	74686415	74687614	-	265	8.88	27968.73	Cell wall
PeEXPA34	PH02Gene38738.t1	17	74771063	74772091	-	262	8.84	27773.77	Cell wall
PeEXPA35	PH02Gene12295.t1	18	13041933	13042833	+	262	5.32	27740.38	Cell wall
PeEXPA36	PH02Gene28555.t1	21	106668295	106669276	-	255	7.43	27252.88	Cell wall
PeEXPA37	PH02Gene28554.t1	21	106691612	106692324	-	170	8.08	18011.44	Cell wall
PeEXPA38	PH02Gene28553.t1	21	106708256	106709246	-	259	7.48	27127.36	Cell wall
PeEXLA6	PH02Gene00824.t1	21	109570927	109572618	-	275	7.47	29170.17	Cell wall
PeEXPB20	PH02Gene31719.t1	21	113342418	113343303	-	224	5.96	23893.25	Cell wall
PeEXPB21	PH02Gene29554.t1	21	113595397	113596188	-	239	5.64	25635.34	Cell wall
PeEXPB22	PH02Gene29555.t1	21	113599967	113600758	-	239	5.64	25635.34	Cell wall
PeEXPB23	PH02Gene29556.t1	21	113617586	113618377	-	239	5.64	25635.34	Cell wall
PeEXPB24	PH02Gene29557.t1	21	113621023	113621814	-	239	6.43	25591.34	Cell wall
PeEXPB19	PH02Gene32845.t3	21	34740938	34745733	-	383	5.24	38728.73	Cell wall
PeEXPA39	PH02Gene06071.t1	21	4378935	4381276	+	264	9.39	27840.66	Cell wall
PeEXPA40	PH02Gene47499.t1	21	61681859	61682843	-	254	9.00	26600.03	Cell wall
PeEXPA41	PH02Gene12421.t1	21	70119688	70120716	-	252	8.77	26166.42	Cell wall
PeEXPA42	PH02Gene02332.t1	23	14914198	14917558	-	261	8.59	27577.60	Cell wall
PeEXPB25	PH02Gene09763.t1	23	23202371	23205955	-	278	8.32	29244.56	Cell wall
PeEXPA43	PH02Gene10678.t1	23	64135918	64136876	-	263	8.78	27857.41	Cell wall
PeEXPB26	PH02Gene02089.t2	24	51338576	51342162	+	278	7.53	29187.25	Cell wall
PeEXPB27	PH02Gene20831.t1	24	54365891	54367152	+	266	5.25	26921.25	Cell wall
PeEXPB28	PH02Gene20830.t1	24	54374823	54375982	+	281	8.84	28391.10	Cell wall
PeEXPA44	PH02Gene00624.t1	24	58890492	58893457	+	261	8.38	27660.73	Cell wall
PeEXLA7	PH02Gene50483.t1	3160	668	2307	+	280	9.07	29758.95	Cell wall
PeEXPA45	PH02Gene50867.t1	12018	7231	8030	-	208	9.40	22617.49	Cell wall
PeEXPB29	PH02Gene51490.t1	17722	23	1312	-	245	8.93	26608.53	Cell wall

Table S2. Detailed primer sequences for quantitative real-time PCR analysis

Gene Name	Forward primer(5'-3')	Reverse primer(5'-3')
PeEXPA1	TCCACCACTACCACCACCTTT	GCGGAGGAATCGAACGAA
PeEXPA3	GGCACGGCAACTTACATCAA	AGGCACCAGAGGATGTTGTT
PeEXPA6	AACGGCCACTCCTACTTCAA	GTCCAGGTACGAGTTGCTCT
PeEXPA11	GAGACAATCGCCATCTACCAG	GCCGACGTTTGAAACCAG
PeEXPA17	TAGCAGCAGCCAGCAAGT	AACAACAAACGGCACAGC
PeEXPA19	ACTTCTGCCCGCCCAACT	ATCCCGCCCTTCTTCACG
PeEXPA20	TCCTACTTCAACCTGGTGCTGG	CGTTGTACGACACGACGGAGC
PeEXPA29	AGAAACAGTGAAGCCACATG	GCTCAGAAACACGACCAAG
PeEXPA31	CACCAACTTCTGCCCGCCAAAC	ACCGGATGCCGCCCTTCTTC
PeEXPA41	CCTCACCTGCGCCAACGT	GCTGGACGGGTCAATCCT
PeEXPA44	TGTGCGGTTCTCCGTCTCA	ATTGGTCAGCGTCGTGCC
PeEXPB3	CGGTGTCAGAAGAGGAGG	AAGGATTGCGTGGTAAAA
PeEXPB5	CGCTGACTGCTCCGATAGAG	ACACCACTTGCCTCCTCCAT
PeEXPB27	AAGGGCTGTGGCTCTTGCT	CACGGGTGTATTGGATTTGGA
PeEXPB28	GCCGCGTTAGAAGAGGAG	ACCGCAAAGCATAAGGTTGT
PeEXPB29	CGGCTCTTGCTACGAGATAAA	TACTTGCACCGCACCCTC
PeEXLA2	ACATCTGGGCTTCACTTTCTGC	GCTCGTCCCTTCCACTCTTACT
PeEXLA5	CGGTTCTTGCGGGTATGG	AGATCGGTGCGGTTCGTC
PeEXLA6	TTCTCCTCCTCCCTTTTGCT	AGGAAGCCATCGTTGAAGGA
